# Multivalent HA DNA Vaccination Protects against Highly Pathogenic H5N1 Avian Influenza Infection in Chickens and Mice

**DOI:** 10.1371/journal.pone.0002432

**Published:** 2008-06-18

**Authors:** Srinivas Rao, Wing-Pui Kong, Chih-Jen Wei, Zhi-Yong Yang, Martha Nason, Darrel Styles, Louis J. DeTolla, Erin M. Sorrell, Haichen Song, Hongquan Wan, Gloria C. Ramirez-Nieto, Daniel Perez, Gary J. Nabel

**Affiliations:** 1 Vaccine Research Center, National Institute of Allergy and Infectious Diseases, National Institutes of Health, Bethesda, Maryland, United States of America; 2 United States Department of Agriculture, Animal and Plant Health Inspection Service, Riverdale, Maryland, United States of America; 3 College of Veterinary Medicine, University of Maryland, College Park, Maryland, United States of America; 4 Comparative Medicine, University of Maryland Baltimore, Baltimore, Maryland, United States of America; University of California Merced, United States of America

## Abstract

**Background:**

Sustained outbreaks of highly pathogenic avian influenza (HPAI) H5N1 in avian species increase the risk of reassortment and adaptation to humans. The ability to contain its spread in chickens would reduce this threat and help maintain the capacity for egg-based vaccine production. While vaccines offer the potential to control avian disease, a major concern of current vaccines is their potency and inability to protect against evolving avian influenza viruses.

**Methodology / Principal Findings:**

The ability of DNA vaccines encoding hemagglutinin (HA) proteins from different HPAI H5N1 serotypes was evaluated for its ability to elicit neutralizing antibodies and to protect against homologous and heterologous HPAI H5N1 strain challenge in mice and chickens after DNA immunization by needle and syringe or with a pressure injection device. These vaccines elicited antibodies that neutralized multiple strains of HPAI H5N1 when given in combinations containing up to 10 HAs. The response was dose-dependent, and breadth was determined by the choice of the influenza virus HA in the vaccine. Monovalent and trivalent HA vaccines were tested first in mice and conferred protection against lethal H5N1 A/Vietnam/1203/2004 challenge 68 weeks after vaccination. In chickens, protection was observed against heterologous strains of HPAI H5N1 after vaccination with a trivalent H5 serotype DNA vaccine with doses as low as 5 µg DNA given twice either by intramuscular needle injection or with a needle-free device.

**Conclusions/Significance:**

DNA vaccines offer a generic approach to influenza virus immunization applicable to multiple animal species. In addition, the ability to substitute plasmids encoding different strains enables rapid adaptation of the vaccine to newly evolving field isolates.

## Introduction

The highly pathogenic H5N1 influenza virus causes lethal multi-organ disease in poultry, resulting in significant economic losses and a public health concern in many parts of the world. The greatest threats posed by this virus are its ability to cause mortality in humans, its potential to compromise food supplies, and its possible economic impacts. Viral maintenance in poultry potentiates the risk of human-to-human transmission and the emergence of a pandemic strain through reassortment. An effective, safe poultry vaccine that elicits broadly protective immune responses to evolving flu strains would provide a countermeasure to reduce the likelihood of transmission of this virus from domestic birds to humans and simultaneously would protect commercial poultry operations and subsistence farmers.

DNA vaccines have been shown to elicit robust immune responses in various animal species, from mice to nonhuman primates [1]–[11]. In human trials, these vaccines elicit cellular and humoral immune responses against various infectious agents, including influenza, SARS, SIV and HIV. In addition to their ability to elicit antibody responses, they also stimulate antigen-specific and sustained T cell responses [1]–[3], [6], [12], [13]. DNA vaccination has been used experimentally against various infectious agents in a variety of mammals, including cattle (against infectious bovine rhinotracheitis/bovine diarrhea virus, leptospirosis and mycobacteriosis) [14], [15], pigs (against classical swine fever virus and mycoplasmosis) [16], and horses (against West Nile virus and rabies) [17]. In addition, DNA vaccines have been tested against avian plasmodium infection in penguins [18] and against influenza and infectious bursal disease in chickens [7], [8], [19], duck hepatitis B virus in ducks [6], and avian metapneumovirus and *Chlamydia psittaci* in turkeys [20], [21] (reviewed in ref. [22]). While they have been used in chickens to generate antisera to specific influenza viruses and confer protection against the low pathogenicity H5N2 strain [23], there is only one previous report of a monovalent DNA vaccine effective against H5N1 (and that only against a matched H5N1 isolate) [24]; no protection with multivalent DNA vaccines against heterologous strains has been reported.

Development and characterization of a DNA vaccine modality for use in poultry offers a potential countermeasure against HPAI H5N1 avian influenza outbreaks. The virus can infect humans, typically from animal sources, including commercial and wild avian species, livestock, and possibly other non-domesticated animal species [25]–[27]. While there is marked diversity in the host range of type A influenza viruses, many experts have speculated that a pandemic strain of type A influenza could evolve in avian species or avian influenza viruses could contribute virulent genes to a pandemic strain through reassortment [28], [29]. Thus, there is reason to consider vaccination of poultry that would stimulate potent and broad protective immune responses [7], [30], [31]. In undertaking such efforts, it is important that there be a differentiation of infected from vaccinated animals [32] so that animals can be protected and permit monitoring of new infections using proven and sensitive methodologies.

In this study, we used an automated high capacity needle-free injection device, Agro-Jet® (Medical International Technology, Inc., Denver, CO) to explore the feasibility of DNA vaccination of poultry. After optimization of injection conditions, alternative multivalent DNA vaccine regimens were analyzed and compared for magnitude and breadth of neutralizing antibodies, as well as protective efficacy after challenge in mouse and chicken models of HPAI H5N1 infection. The findings suggest that it is possible to develop a multivalent DNA vaccine for poultry that can protect against multiple HPAI H5N1 strains and that could keep pace with the continued evolution of avian influenza viruses.

## Results

### Immunogenicity and neutralizing antibody specificity of alternative HA DNA vaccines in mice

**Figure 1:**
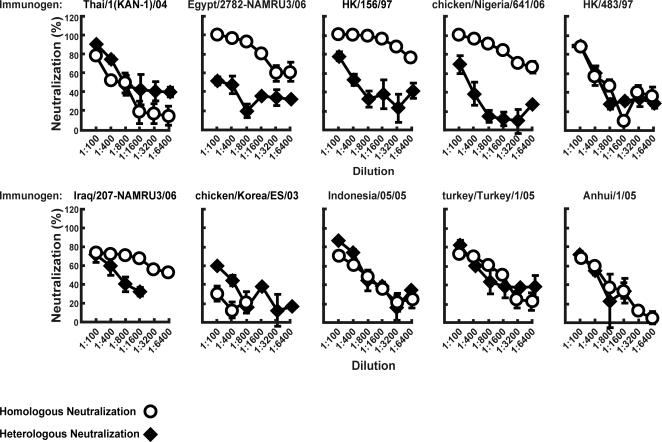
Sera from mice immunized with plasmid DNA vaccines encoding HA from specific strains neutralize a homologous and a heterologous HA with differing efficacy. Groups of mice (n = 10) were immunized as described in Materials and Methods with 15 µg of individual H5 HA DNA expression vectors, pCMV/R 8κB, encoding the HA of indicated viruses: A/Indonesia/05/2005, A/Anhui/1/2005, A/Thailand/1(KAN-1)/2004, A/Hong Kong/156/1997, A/Hong Kong/483/1997, A/chicken/Korea/ES/2003, A/turkey/Turkey/1/2005, A/Egypt/2782-NAMRU3/2006, A/chicken/Nigeria/641/2006, and A/Iraq/207-NAMRU3/2006. Sera was collected from each group 14 days after the third immunization, pooled, and tested against the homologous (open circles) or a heterologous HA, A/Vietnam/1203/2004 (black diamonds). Serum from each group was serially diluted (1∶100 to 1∶6400) and analyzed by LAI. Error bars at each point indicate the standard deviation; each sample was evaluated in triplicate. Different degrees of neutralization among various H5 pseudoviruses were observed among different HA-immunized mice.

To evaluate the efficacy of multivalent DNA vaccines, initial studies were performed in mice. Expression vectors encoding HAs from ten phylogenetically diverse strains of influenza viruses [33] were generated by synthesis of cDNAs (see Materials and Methods) in plasmid expression vectors, pCMV/R or pCMV/R 8κB, which mediates high level expression and immunogenicity *in vivo* [34], [35], [36]. Animals were immunized with each expression vector intramuscularly (IM) at three week intervals, and the antisera were evaluated on day 14 after the third immunization for their ability to neutralize HPAI H5N1 pseudotyped lentiviral vectors as previously described [35], [36]. We have previously shown that lentiviral assay inhibition (LAI) yields similar results to microneutralization and HAI analyses with higher sensitivity in mice [35], [36]. Significant neutralizing antibodies generated to homologous HAs were detected consistently by LAI with few exceptions, while cross reactivity to a standard isolate, A/Vietnam/1203/2004, was variable. For example, IC90 titers exceeding 1∶800 were observed against A/chicken/Nigeria/641/2006 and A/Hong Kong/156/1997, while a lesser response was detected for the A/chicken/Korea/ES/2003 strain (Fig. 1). Heterologous neutralization to A/Vietnam/1203/2004 was variable and did not fully correlate with the degree of relatedness of the specific HA. The ability of these immunogens to generate robust cross-reactive antibodies is consistent with previous observations [4], [37], [38].

### Multivalent HA vaccination response in mice

**Figure 2:**
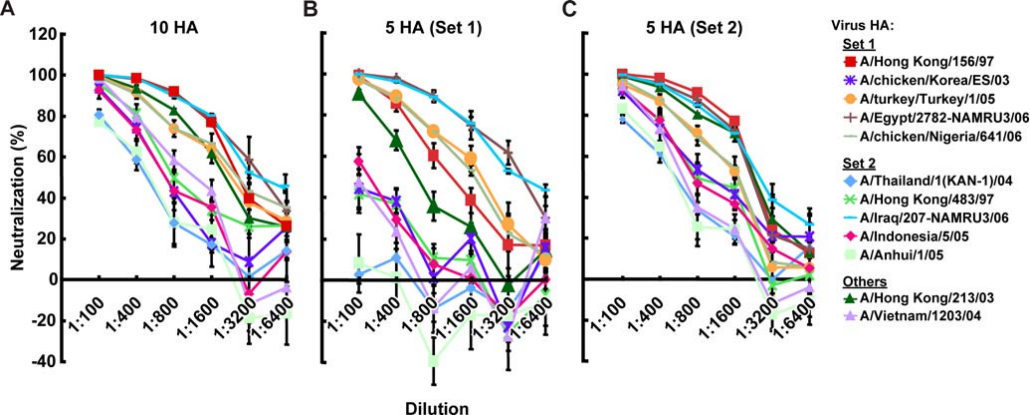
Breadth of neutralization against divergent HAs is determined by the composition of multivalent HA plasmid DNA expression vectors. Neutralization of 12 different H5N1 isolates was determined in the LAI assay using serum from mice immunized with (A) 10 HAs comprised of: pCMV/R 8κB-HA(A/Hong Kong/156/1997), pCMV/R 8κB-HA(A/chicken/Korea/ES/2003), pCMV/R 8κB-HA(A/turkey/Turkey/1/2005), pCMV/R 8κB-HA(A/Egypt/2782-NAMRU3/2006), pCMV/R 8κB-HA(A/chicken/Nigeria/641/2006), pCMV/R 8κB-HA(A/Indonesia/05/2005), pCMV/R 8κB-HA(A/Anhui/1/2005), pCMV/R 8κB-HA(A/Thailand/1(KAN-1)/2004), pCMV/R 8κB-HA(A/Hong Kong/483/1997), and pCMV/R 8κB-HA(A/Iraq/207-NAMRU3/2006) as in 5 HA (Set 1) plus in 5 HA (Set 2). (B) 5 HA (Set 1) composed of vectors: pCMV/R 8κB-HA(A/Hong Kong/156/1997), pCMV/R 8κB-HA(A/chicken/Korea/ES/2003), pCMV/R 8κB-HA(A/turkey/Turkey/1/2005), pCMV/R 8κB-HA(A/Egypt/2782-NAMRU3/2006), and pCMV/R 8κB-HA(A/chicken/Nigeria/641/2006)., or (C) 5 HA (Set 2) contained: pCMV/R 8κB-HA(A/Indonesia/05/2005), pCMV/R 8κB-HA(A/Anhui/1/2005), pCMV/R 8κB-HA(A/Thailand/1(KAN-1)/2004), pCMV/R 8κB-HA(A/Hong Kong/483/1997), and pCMV/R 8κB-HA(A/Iraq/207-NAMRU3/2006). Mice were vaccinated as described in Materials and Methods. In this experiment, the DNA vaccine consisted of 10 plasmids (1.5 µg each) expressing HA proteins as indicated. In panels B and C, mice (n = 10) were immunized with 15 µg of plasmid (3 µg each) three times at 3 week intervals. Serum pools from the immunized animals were collected 14 days after the third immunization. The antisera were tested against the 12 indicated pseudotyped lentiviral vectors at varying dilutions. Error bars at each point indicate the standard deviation; each sample was evaluated in triplicate. In general, the immunized serum neutralized all tested pseudotyped lentiviruses at low dilutions while differences were often observed at high dilution.

To determine whether immunization with multiple HAs simultaneously could expand the breadth of the neutralizing antibody response without significant loss of magnitude, a combination of 10 HA DNA vaccine immunogens was administered IM at proportionally lower concentration (1.5 µg per immunogen) into groups of 10 mice (see Materials and Methods). Remarkably, despite a log lower DNA concentration of each component, significant neutralizing antibody titers were generated to each of the 10 immunogens, with >80% neutralization against 6 out of 12 H5 HA pseudoviruses at dilutions of up to 1∶400 (Fig. 2A).

To evaluate whether similar breadth of immunity could be generated with fewer immunogens, two different combinations of 5 immunogens were selected, based on the phylogenetic diversity of HA among the avian influenza viruses [33] and the cross-reactivity of the neutralizing antibody responses of select individual immunogens (Fig. 1). As expected, there were substantial differences in the breadth of neutralization between these two sets of 5 immunogen multivalent vaccines (Fig. 2, B vs. C). In one set, while neutralization of homologous strains was comparable to the monovalent and the 10 immunogen multivalent immune response, fewer cross-reactive antibodies were detected, directed most prominently against A/Iraq/207-NAMRU3/2006 and A/Egypt/ 2782-NAMRU3/2006 (Fig. 2B; 5 HA, Set 1). In contrast, set 2 elicited consistent and comparable neutralization against both homologous and heterologous viruses at titers exceeding 1∶400 against most of the tested HA strains (Fig. 2C; 5 HA, Set 2), as observed in the 10 component multivalent DNA vaccine. It was therefore possible to use multivalent DNA immunization and selection based on neutralizing antibody breadth and phylogenetic relationships to reduce the number of components in the vaccine while maintaining substantial breadth of cross neutralization.

### Protection of DNA-vaccinated mice against challenge with heterologous H5N1 A/Vietnam/1203/2004 influenza virus

**Figure 3:**
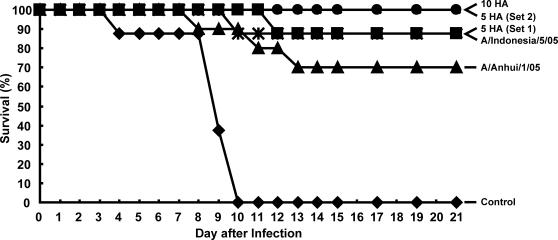
Immune protection conferred against lethal challenge of A/Vietnam/1203/2004 in mice 68 weeks after vaccination. Mice were immunized with 15 µg total of either the 10 HA as in Fig. 2A, legend and Materials and Methods, 5 HA (Set 1) as in Fig. 2B, legend and Materials and Methods, 5 HA (Set 2) as mentioned in Fig. 2C, legend and Materials and Methods, monovalent A/Indonesia/05/2005 HA, monovalent A/Anhui/1/2005 HA, or Control (empty vector) three times at three week intervals as described in Fig. 2, legend. Animals (n = 8–10 per group) were challenged 68 weeks later by intranasal inoculation. All control mice died 10 days after infection.

Mice immunized as described above were challenged with a heterologous H5N1 virus 68 weeks after the final DNA vaccination. Animals were then challenged with 10 LD_50_ of the highly pathogenic A/Vietnam/1203/2004 virus intranasally, and morbidity and mortality were monitored for 21 days after the viral challenge. The control animals, injected with the plasmid expression vector with no insert, died within 10 days of infection. Complete survival was observed in the groups immunized with the 10 component and set 2 of the 5 component multivalent DNA vaccines (Fig. 3). Immunization with HA derived from the A/Indonesia/05/2005 strain or set 1 of the 5 component multivalent DNA vaccine showed a survival rate approaching 90%. In contrast, animals injected with HA plasmid DNA derived from A/Anhui/1/2005, which has diverged more from A/Vietnam/1203/2004, showed a lower percent survival (70%) after lethal viral challenge. Survival differences between groups were assessed using a log-rank test and the Gehan-Wilcoxon test on the survival curves for pairs of groups. A test was deemed significant if the p-value was <0.01. Mice injected IM with different HAs, A/Indonesia/5/05, A/Anhui/1/05, 10HA, 5 HA (Set 1), or 5 HA (Set 2) showed a significant difference compared to control (all p values<0.001). Among the HA-immunized groups, there was no significant difference between any two groups (p>0.08 for all comparisons). For example, no significant difference was observed between the A/Anhui/1/05 group, which had the least survival among the HA immunized groups (7 out of 10), and other HA groups: A/Indonesia/5/05 (p = 0.377), 10 HA (p = 0.082), 5 HA (Set 1) (p = 0.101), or 5 HA (Set 2) (p = .411). Therefore, we cannot exclude the possibility that the 3 deaths in the A/Anhui/1/05 group may have been due to random chance.

### Neutralizing antibody responses in chickens after HA DNA immunization

Since it is desirable to confer protective immunity in poultry and HA DNA vaccination was effective in mice, we next examined the breadth and potency of single or multiple HA plasmid immunization in chickens. The ability of chickens to generate specific antibodies was assessed with three strains that showed broad cross protection in mouse studies (A/Vietnam/1203/2004, A/Anhui/1/2005 and A/Indonesia/05/2005), administered individually or in combination, by different injection methods. In addition to needle injection, a needle-free repetitive injection device, Agro-Jet® (Medical International Technology, Inc., Denver, CO), was analyzed. This device disperses the 0.1 to 5 ml injection doses into the dermal, subcutaneous, or intramuscular tissue depending upon the pressure adjustments, powered by a CO_2_ gas pressure plunger [39]. The injection conditions were determined by histologic analysis of tissues that received injections of India ink; a pressure of 48 psi was chosen since it enabled consistent delivery into intradermal and subcutaneous tissues (Fig. S1).

**Figure 4:**
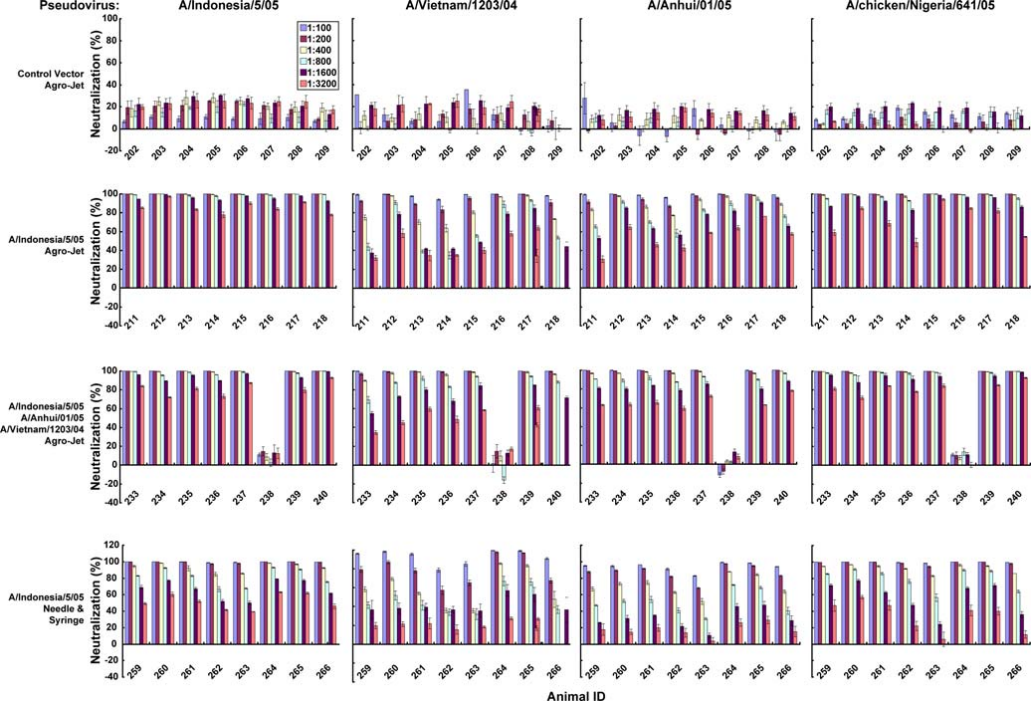
Neutralizing antibody responses against homologous and heterologous HAs from chickens immunized with HA expression vectors by different routes. Neutralization against the indicated strain HAs was analyzed after immunization with trivalent HA encoding plasmids: pCMV/R-HA(A/Indonesia/05/2005), pCMV/R-HA(A/Anhui/1/2005), and pCMV/R-HA(A/Vietnam/1203/2004) with the indicated delivery device using sera taken two weeks after the third injection. Neutralization was determined by LAI from individual chickens at titers ranging from 1∶100 to 1∶3200. Bird #238 consistently showed a low level of neutralization, possibly because of an inhibitor in the serum because it was fully protected against viral challenge. Percent neutralization was calculated by the reduction of luciferase activity relative to the values achieved in the non-immune sera.

Immunization of chickens with the control plasmid (CMV/R) without an HA gene insert elicited minimal neutralizing antibody titers compared to HA-immunized animals 1 week after 3 DNA immunizations. Nearly all chickens immunized with either monovalent or multivalent HA DNA vaccines generated significant neutralization titers (Fig. 4 and Table S1). In general, there was a progressive increase in the amount of neutralization after each successive DNA vaccination (data not shown) with maximal response at 1 week after the 3^rd^ DNA immunization, with highest and most consistent levels in the trivalent vaccine group delivered with the Agro-Jet® device. Neutralization of the Indonesia HA strain was the most robust, with neutralization nearing 100% at titers greater than 1∶3200. Both the monovalent and multivalent vaccines elicited robust homologous (Fig. 4, A/Indonesia/05/2005) and heterologous HA neutralization (Fig. 4, A/Nigeria/641/05). Similar responses were noted in the other groups, including administration of monovalent HA-A/Indonesia/05/2005 subcutaneously by needle syringe and via Agro-Jet® (Fig. 4, second row). The neutralization response of chickens immunized with monovalent Indonesia was similar to that of the trivalent vaccine(Fig. 4). Even though one chicken (238) in the multivalent vaccine group produced almost the same degree of neutralization at each time point and was protected, it did not produce a high neutralizing antibody titer for reasons that were uncertain but possibly related to a non-specific inhibitor in the sera.

### Protection of DNA-vaccinated chickens against challenge with A/Vietnam/1203/2004 influenza virus

**Figure 5:**
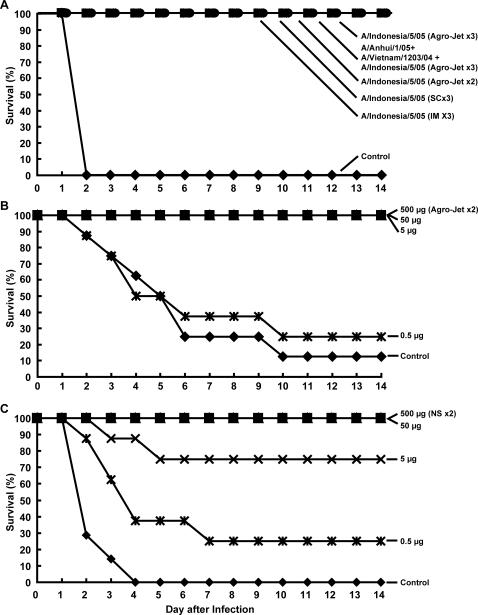
Immune protection after DNA vaccination against lethal challenge with heterologous A/Vietnam/1203/2004 using needle or needle-free injection in chickens and dose-response analysis of efficacy for each route. A. Chickens were immunized with either trivalent HA encoding plasmids: pCMV/R-HA(A/Indonesia/05/2005) plus pCMV/R-HA(A/Anhui/1/2005) plus pCMV/R-HA(A/Vietnam/1203/2004) by Agro-Jet® three times, monovalent plasmid, pCMV/R-HA(A/Indonesia/05/2005) with Agro-Jet® twice or three times, or by needle and syringe subcutaneously or IM three times as indicated. Control animals were injected with a no insert vector by needle and syringe IM three times. A total of 500 µg plasmid DNA was used in each injection for all groups. Controls died 2 days after infection by nasal inoculation. The geometric mean reciprocal endpoint titers (GMT) for hemagglutination inhibition (HI) in each group one week after the last vaccination and two weeks post-challenge respectively were: Control: undetectable, not done; Trivalent by Agro-Jet (×3): 777, 304; A/Indonesia/05/2005 by Agro-Jet (×3): 320, 285; A/Indonesia/05/2005 by Agro-Jet (×2): 516, 533; A/Indonesia/05/2005 by needle (subcutaneous ×3): 211, 155; A/Indonesia/05/2005 by Agro-Jet (intramuscular ×3): 118, 495. B. Immunization with trivalent DNA vaccine, pCMV/R-HA(A/Anhui/1/2005), pCMV/R-HA(A/Indonesia/05/2005), and pCMV/R-HA(A/chicken/Nigeria/641/2006), twice by Agro-Jet® intradermally/subcutaneously in different doses (500 µg, 50 µg, 5 µg, and 0.5 µg) as indicated. Controls were immunized with 500 µg empty vector. Controls died 4 days after infection by intranasal inoculation. The GMT HI in each group two weeks post-challenge were: Control: 80 (n = 1); Trivalent by Agro-Jet (500 µg): 580 (n = 8); Trivalent by Agro-Jet (50 µg): 430 (n = 8); Trivalent by Agro-Jet (5 µg): 183 (n = 8); Trivalent by Agro-Jet (0.5 µg): 200 (n = 2). C. Immunization with trivalent DNA vaccine pCMV/R-HA(A/Anhui/1/2005), pCMV/R-HA(A/Indonesia/05/2005), and HA(A/chicken/Nigeria/641/2006) was performed twice by needle and syringe IM at doses outlined in panel B. The GMT HI in each group two weeks post-challenge were: Control: not done (n = 0); Trivalent by needle (500 µg): 325 (n = 8); Trivalent by needle (50 µg): 120 (n = 8); Trivalent by needle (5 µg): 197 (n = 8); Trivalent by needle (0.5 µg): 200 (n = 2). The chickens in panel B and C each received two immunizations.

To determine whether chickens immunized with single or multiple DNA vaccines were protected from a lethal challenge of a heterologous HPAI H5N1 virus, vaccinated chickens were inoculated with 20 LD_50_ of highly pathogenic A/Vietnam/1203/2004 heterologous virus intranasally using standard methods [25], [40] and monitored for morbidity, mortality, viral shedding and serum antibodies. While all the control animals died within 2 days of infection, 100% survival was noted in the rest of the chickens (Fig. 5A). The animals that were healthy, showing no signs of clinical disease or malaise, were euthanized on day 14. There was no evidence for viral shedding monitored via tracheal and cloacal swabs of infected chickens 2–14 days after challenge as determined by embryonal inoculation (data not shown: egg infectious dose 50 (EID_50_) limit of detection ∼100 virus particles).

### Relative potency of DNA vaccines delivered by needle or needle-free injection devices

To compare the relative efficacy of DNA vaccines delivered IM by needle and syringe versus the needle-free Agro-Jet® device injection, a dose-response study was performed with amounts of DNA vaccine ranging from 500 to 0.5 µg with two inoculations. In these experiments, the HA derived from A/chicken/Nigeria/641/2006 was substituted for A/Vietnam/1203/2004 since it represented a more contemporary isolate. The observed rate of protection was higher among the animals receiving 5 µg by Agro-Jet (8/8) than by IM injection (6/8) (Fig. 5, B vs. C). Both modes provided complete protection for all animals at doses higher than this, and 25% protection for the animals receiving 0.5 µg doses (Fig. 5B, C). Survival differences between consecutive doses were assessed using a log-rank test on the survival curves for pairs of groups. A test was deemed significant if the p-value was <.01, and marginally significant if the p-value was <.05 but >.01. Chickens injected IM showed a marginally significant difference between 0.5 and 5 µg (p = .047). In the same group there was a significant difference between control and 5, 50 and 500 µg (p<.001 for all comparisons) and the difference between control and 0.5 µg was marginally significant (p = .016). Chickens that were injected using Agro-Jet® showed a significant difference between 0.5 and 5 µg (p = .004) and between control and 5, 50, and 500 µg (p<.001 for all comparisons). There were no differences between control and 0.5 µg or between 5, 50, and 500 µg. Lastly, the survival differences between Agro-Jet® and IM for each dose group were not significant. The neutralizing antibody response to homologous and heterologous HAs corresponded with protection and correlated with dose, with higher titers elicited by injection with Agro-Jet® compared to needle (Table S2). We assessed viable viral shedding after inoculation by chick embryo inoculation three days after virus challenge (Week 8). While we noted some embryonic lethality at the 0.5 µg dose, there was no embryonic lethality at 5, 50 or 500 µg groups (data not shown).

## Discussion

Since the HPAI H5N1 virus first appeared ten years ago, this highly pathogenic avian influenza virus has shown increasing diversification and dissemination in Asia, Africa, and Europe [28], [41]–[44]. In addition to its effects on human health by cross-species transmission [28], [45], [46] and ability to compromise food sources, it poses a continuing threat to public health as it evolves and adapts in different species. The pandemic potential of this virus, especially as it relates to the poultry industry and for reservoir avian hosts, underscores the need for a vaccine that offers broad spectrum immunity and protection against lethal viral challenge. While the virus remains restricted in its ability to infect humans and undergo efficient human-to-human transmission [28], [47], its persistence and spread in poultry increases the risk of the emergence of a pandemic strain. One approach to pandemic risk reduction is to limit the propagation of the virus in poultry and other relevant avian species.

We have previously reported that DNA vaccines encoding HA can confer protection against a highly lethal human pandemic influenza virus, the 1918 H1N1 virus, in mice [36]. DNA vaccines offer several advantages, including the ability to express diverse antigens, tolerability in various hosts, ease of delivery, and stability for storage and distribution without the necessity of maintaining a cold chain; they have been shown to be safe and efficacious in a variety of animal models [2], [4], [12], [22], [48]. Because they do not contain other viral proteins used to screen for infection, they also address the need to differentiate vaccinated from infected animals. There is evidence that DNA vaccination elicits cell-mediated immunity against influenza HA in addition to inducing an antibody response [36], an effect that could significantly contribute to protective immunity as viruses show genetic drift and reduced susceptibility to neutralization.

Ideally, a highly effective influenza vaccine should not only be able to let the host develop a protective immune response against a matching live virus challenge but also elicit robust protective immune responses against a broad range of homologous and heterologous H5 influenza strains. A multivalent H5 vaccine containing diverse serotypes could expand the antigenic breadth sufficiently to provide protection against heterologous challenge and may preclude the emergence of vaccine-resistant strains that may arise due to evolutionary vaccine pressure on the virus. Due to the antigenic drift and shift of the influenza virus genome, it has been very difficult to predict the next dominant strain of an avian endemic outbreak. DNA vaccines can be synthesized in a relatively short period of time, and the targeted mutations can be tailored to specific viral serotypes. The mutations promote a focused and enhanced immune response [3], [49], [50] that may be particularly important in the event of an outbreak where specificity is the key to epidemic control. The use of modified codons ensures maximal expression in the host and eliminates the possibility of recombination with influenza viruses that might potentially generate new strains.

A more broadly protective murine vaccine was developed here by including more HAs from varying strains in the multivalent vaccine (Figs. 2 and 3). However, it is less practical to include large numbers of different HAs in one vaccine due to the cost and complexity of manufacturing such a vaccine. Therefore, we simplified the vaccine regimen based on cross-neutralization studies and phylogenetic relationships. A trivalent vaccine was subsequently identified for further studies. Due to the availability of the A/Vietnam/1203/200 H5N1 for the animal challenge studies, 3 components of HA including A/Vietnam/1203/2004, A/Indonesia/05/2005, and A/Anhui/1/2005 HA were tested as the vaccine candidate in the challenge study. This trivalent DNA HA vaccine including A/Indonesia/05/2005, A/Anhui/1/2005 and A/Vietnam/1203/2004 or A/chicken/Nigeria/641/2006 HAs included representatives of a broad range of influenza strains by HA sequence analysis [51].

While three DNA immunizations were used initially to demonstrate protective immunity and have been used previously to elicit protection in mice [36], we found that effective protective immunity could be induced with two DNA vaccinations and as little as 5 µg trivalent DNA immunization using the ID/SC route with the Agro-Jet® device. In addition, based on the chick embryo inoculation data, we believe that there is effective neutralization of the virus and lack of infectious viral shedding in chicken vaccinated with as little as 5 µg of DNA. The device's capacity for rapid repetitive injection and the lower quantity and stability of DNA enhance the practicality and utility of this approach for vaccination of endangered species in captivity or administration to poultry or other animals.

## Materials and Methods

### Viruses

A/Vietnam/1203/2004 (H5N1) (A/VN/1203/04) was obtained from the repository at the Centers for Disease Control and Prevention (CDC), Atlanta, Georgia. The virus was propagated in 10-day old embryonated chicken eggs at 35°C and stored at −70°C until use. The virus was titrated by the Reed and Muench method to determine EID_50_ [52].

### Immunogen and plasmid construction

Plasmids encoding different versions of H5 HA protein (A/Thailand/1(KAN-1)/2004 (clade 1) GenBank AY555150; A/Vietnam/1203/2004 (clade 1) GenBank AY651334; A/Hong Kong/156/1997 (clade 0) GenBank AAC32088; A/Hong Kong/483/1997 GenBank AAC32099.1(clade 0); A/chicken/Korea/ES/2003 (clade 2.5) GenBank AAV97603.1; A/Indonesia/05/2005 (clade 2.1.3) ISDN125873; A/turkey/Turkey/1/2005 (clade 2.2) GenBank DQ407519; A/Egypt/2782-NAMRU3/2006 (clade 2.2) GenBank ABE01046; A/chicken/Nigeria/641/2006 (clade 2.2) GenBank DQ406728; A/Iraq/207-NAMRU3/2006 (clade 2.2) GenBank DQ435202; A/Anhui/1/2005 (clade 2.3.4) GenBank ABD28180) were synthesized using human-preferred codons (GeneArt, Regensburg, Germany) [36]. HA cDNAs from diverse strains of influenza viruses were then inserted into plasmid expression vectors, pCMV/R or pCMV/R 8κB, which mediates high level expression and immunogenicity *in vivo* [34], [35], [36]. For initial trivalent immunizations in chickens, the A/Vietnam/1203/2004, A/Anhui/1/2005 and A/Indonesia/05/2005 strains were used and in the dose response study, the Vietnam strain was replaced with A/chicken/Nigeria/641/2006. The immunogens used in DNA vaccination contained a cleavage site mutation (PQRERRRKKRG to PQRETRG) as previously described [35], [36]. This mutation was generated by site-directed mutagenesis using a QuickChange kit (Stratagene, La Jolla, CA).

### DNA immunization of mice

6–8 week old female BALB/c mice were purchased from The Jackson Laboratory and maintained in the AAALAC-accredited Vaccine Research Center Animal Care Facility (Bethesda, MD) under specific pathogen-free conditions. All experiments were approved by the Vaccine Research Center Animal Care and Use Committee. The mice were immunized as previously described [5]. Briefly, mice (10 animals for all test groups, 20 animals for the negative control group) were immunized three times with a total of 15 µg plasmid DNA in 100 µl of PBS (pH 7.4) IM at weeks 0, 3 and 6. For the single plasmid groups, the DNA plasmid in a volume of 100 µl was administered to each animal: pCMV/R 8κB, pCMV/R 8κB-HA(A/Indonesia/05/2005), pCMV/R 8κB-HA(A/Anhui/1/2005), pCMV/R 8κB-HA(A/Thailand/1(KAN-1)/2004), pCMV/R 8κB-HA(A/Hong Kong/156/1997), pCMV/R 8κB-HA(A/Hong Kong/483/1997), pCMV/R 8κB-HA(A/chicken/Korea/ES/2003), pCMV/R 8κB-HA(A/turkey/Turkey/1/2005), pCMV/R 8κB-HA(A/Egypt/2782-NAMRU3/2006), pCMV/R 8κB-HA(A/chicken/Nigeria/641/2006), and pCMV/R 8κB-HA(A/Iraq/207-NAMRU3/2006). The 10 plasmid combination group (10 HA) received 1.5 µg DNA for each of the 10 HA plasmids (total 15 µg) as used in the single plasmid groups mentioned above. For the two 5 plasmid combination groups [5 HA (Set1), 5 HA(Set 2)], 3 µg of each plasmid DNA were used as total DNA remained the same (15 µg). 5 HA (Set 1) group contained: pCMV/R 8κB-HA(A/Hong Kong/156/1997), pCMV/R 8κB-HA(A/chicken/Korea/ES/2003), pCMV/R 8κB-HA(A/turkey/Turkey/1/2005), pCMV/R 8κB-HA(A/Egypt/2782-NAMRU3/2006), and pCMV/R 8κB-HA(A/chicken/Nigeria/641/2006). 5 HA (Set 2) group contained: pCMV/R 8κB-HA(A/Indonesia/05/2005), pCMV/R 8κB-HA(A/Anhui/1/2005), pCMV/R 8κB-HA(A/Thailand/1(KAN-1)/2004), pCMV/R 8κB-HA(A/Hong Kong/483/1997), and pCMV/R 8κB-HA(A/Iraq/207-NAMRU3/2006). Serum was collected 10 days after the last vaccination.

### DNA immunization of chickens

The study was carried out in the AAALAC-accredited animal facility at the University of Maryland School of Medicine. Six groups of 8 one-day-old male and female SPAFAS White Leghorn Chickens, *Gallus domesticus*, were obtained from Charles River Laboratories (Connecticut). The animals were housed in brooder and grower cages (McMurray Hatcheries, Iowa). Feed (Teklad Japanese Quail Diet – 3050, Harlan-Teklad, WI) and water were provided to the animals *ad libitum.* The study was performed in strict accordance with the “Guide” after approvals from the Animal Care and Use Committees of the Vaccine Research Center, NIH and the University of Maryland. DNA immunizations were performed as described at 0, 3 and 6 weeks. A total dose of 500 µg of one or a combination of the following DNA plasmids in a volume of 250 µl was administered to each animal: pCMV/R, pCMV/R-HA(A/Indonesia/05/2005), pCMV/R-HA(A/Anhui/1/2005), and pCMV/R-HA(A/Vietnam/1203/2004). Groups 1–4 received intradermal/subcutaneous immunizations via Agro-Jet® with pCMV/R, with pCMV/R-HA(A/Indonesia/05/2005), with pCMV/R-HA(A/Indonesia/05/2005) plus pCMV/R-HA(A/Anhui/1/2005) plus pCMV/R-HA(A/Vietnam/1203/2004), or with pCMV/R-HA(A/Indonesia/05/2005) respectively. Group 5 received subcutaneous immunizations via needle and syringe with pCMV/R-HA(A/Indonesia/05/2005); and Group 6 received intramuscular immunizations via needle and syringe with pCMV/R-HA(A/Indonesia/05/2005). Blood samples were collected from the alar veins of the chickens at various intervals. All groups were challenged at week 8 intranasally with 5×10^6^ EID_50_/ml of A/Vietnam/1203/2004 H5N1 viruses. For the virus load study, cloacal and tracheal swabs were collected from each animal on days 3 and 5 post challenge and titrated for virus infectivity in embryonated eggs. Chickens were monitored each day for signs of disease or death. Surviving chickens underwent necropsy on day 14 post challenge.

For dose response experiments, five groups were immunized with the trivalent HA vaccine (pCMV/R-HA(A/Anhui/1/2005), pCMV/R-HA(A/Indonesia/05/2005), and pCMV/R-HA(A/chicken/Nigeria/641/2006) using 500 µg (167 µg of each of the three plasmids), 50 µg (17 µg of each plasmid), 5 µg (1.7 µg of each plasmid), 0.5 µg (0.17 µg of each plasmid) and a 500 µg control vector administered IM with needle and syringe, and an additional five groups were injected with the same plasmid doses using the Agro-Jet® injection device.

### Agro-Jet® needle-free injector

Agro-Jet® is a needle-free device used for mass delivery of vaccines and drugs in livestock and poultry. The device is semi-automatic and requires a small CO_2_ tank or compressed air for low pressure delivery. Upon trigger activation, CO_2_ disperses the injectate at a precise dose into the muscle, dermis or subcutaneous tissue depending on the setting that was standardized for our use. We used an effective volume of 0.1 ml in our injectate [39]. In this study we were able to effectively deliver 0.1 ml of injectate into the animal's dermis/subcutaneous tissue at a pressure of 48–55 psi.

### Challenge studies in mice

Sixty-eight weeks after the last immunization, female BALB/c mice were lightly anesthetized with Ketamine/Xylazine and inoculated intranasally with 10 LD_50_ of A/Vietnam/1203/2004 virus diluted in phosphate-buffered saline in a 50 ul volume. Mice were monitored daily for morbidity and measured for weight loss and mortality for 21 days post infection. Any mouse that had lost more than 25% of its body weight was euthanized. All experiments involving the HPAI virus were conducted in an AAALAC accredited facility (BioQual Inc., Gaithersburg, MD) under BSL 3 conditions that included enhancements required by the USDA and the Select Agent Program.

### Challenge studies in chickens

White Leghorn chickens were challenged one week after the last immunization with 20 lethal dose 50 (LD_50_) of A/Vietnam/1203/04 (H5N1) influenza A virus, equivalent to 2×10^4^ EID_50_ based on previous challenges [53]. Chickens were infected with 200 µl virus intranasally. Tracheal and cloacal swabs were collected days 3 and 5 post-challenge and stored in glass vials containing BHI medium (BBL™ Brain Heart Infusion, Becton Dickinson) at −80°C. Blood was collected 14 days post-challenge and serum was titered by microneutralization assay. Chickens were observed and scored daily for clinical signs of infection, morbidity and mortality. Chickens that survived the study were bled and humanely euthanized at day 14 post-challenge. Lungs, heart, intestine and kidney were collected and samples were stored in formalin for histopathology. Experiments were carried out under BSL3+ conditions with investigators wearing appropriate protective equipment and compliant with all Institutional Animal Care and Use Committee-approved protocols and under Animal Welfare Act regulations at the University of Maryland, College Park, Maryland.

### Virus titers in chickens

Representative tracheal and cloacal swabs were chosen to run an EID_50_ assay for comparison and virus titers were determine by the method of Reed and Meunch [52]. Briefly, swabs were used to infect 10 day-old embryonated chicken eggs in 10-fold dilutions. Three eggs were inoculated per dilution and incubated for 48 hours before titration.

### Microneutralization assays

Neutralizing antibodies were titrated from serum samples collected week 5 and 7 post-vaccination and day 14 post-challenge. The microneutralization assay was performed using a 96-well plate format. Serum was treated with receptor-destroying enzyme (Denka Seiken Co.) and treated at 37°C per the manufacturer's instructions. After an overnight incubation and subsequent inactivation samples were brought to a final dilution of 1∶10 using PBS and each sample was serially diluted and virus, diluted to 100 TCID_50_, was added to each well. The plates were then incubated at 37°C, 5% CO_2_ for 1–2 hours. Following incubation, supernatants were used to infect a second 96-well plate of MDCK cells. Microplates were incubated at 4°C for 15 minutes and then 37°C, 5% CO_2_ for 45 minutes. Supernatants of serum and virus were then discarded and 200 µl of OptiMEM (containing 1X antibiotics/antimycotics, 1 µg/ml TPCK-trypsin) was added and incubated at 37°C, 5% CO_2_ for 3 days. After 3 days, 50 µl of the supernatant from each well was transferred into a new 96-well microplate, and an HA assay was performed to calculate the antibody titers. Virus and cell controls were included in the assay.

Two-fold dilutions of heat-inactivated sera were tested in a microneutralization assay as previously described [54] for the presence of antibodies that neutralized the infectivity of 100 TCID_50_ (50% tissue culture infectious dose) of the A/Vietnam/1203/2004 H5N1 virus on MDCK cell monolayers by using two wells per dilution on a 96-well plate.

### Production of pseudotyped lentiviral vectors and measurement of neutralizing antibodies by LAI

The recombinant lentiviral vectors expressing a luciferase reporter gene were produced as previously described [35], [36]. For the neutralization assay, antisera from immunized animals were heat-inactivated at 55°C for 30 minutes and mixed with 50 µl of pseudovirus at various dilutions. The sera/virus mixture was then added to 293A cells in 96-well B&W TC Isoplates (Wallac, Turku, Finland; 12,000 cells/well). Two hours later, the plates were washed and fresh medium was added. Cells were lysed in mammalian cell lysis buffer (Promega, Madison, WI) 24 hrs after infection and luciferase activity was measured using the Luciferase Assay System (Promega, Madison, WI).

The following strains were used for the production of pseudotyped viruses: for HA we used A/Thailand/1(KAN-1)/2004; A/Vietnam/1203/2004; A/Hong Kong/156/1997; A/Hong Kong/483/1997; A/chicken/Korea/ES/2003; A/Indonesia/05/2005; A/turkey/Turkey/1/2005; A/Egypt/2782-NAMRU3/2006; A/chicken/Nigeria/641/2006; A/Iraq/207-NAMRU3/2006; A/Anhui/1/2005, and for N1 NA we used A/Thailand/1(KAN-1)/2004.

### Hemagglutination (HA) and hemagglutination inhibition (HI) assays

The HA/HI titers were determined as previously described [54]. Briefly, HA titers were calculated using 50 µl of 0.5% chicken red blood cell suspension in PBS added to 50 µl of two-fold dilutions of virus in PBS. This mix was incubated at room temperature for 30 minutes. The HA titers were calculated as the reciprocal value of the highest dilution that caused complete hemagglutination. HI titers were calculated by titrating 50 µl of antiserum treated with receptor-destroying enzyme and an equivalent amount of A/Vietnam/1203/2004 virus (four hemagglutinating doses) was added to each well. Wells were incubated at room temperature for 30 minutes and 50 µl of a 0.5% suspension of chicken red blood cells was added. HI titers were calculated after 30 minutes as the reciprocal of the serum dilution that inhibited hemagglutination.

## Supporting Information

Table S1 Hemagglutination inhibition (HI), microneutralization titer (NT), and LAI of sera from individual chickens immunized with different vaccines. Sera from immunized animals were obtained at week 5 or 7, a week before or after the final boost, and neutralization was assessed by HI, microneutralization (NT) and LAI (shown as IC_50_). Individual animal serum of each group is shown and was analyzed as described in the Materials and Methods section.
(0.12 MB DOC)

Table S2 Neutralizing antibody responses after two vaccinations at different doses by LAI. Sera obtained at week 5, one week after the final boost, from individual animals immunized with trivalent DNA HA encoding vaccine: pCMV/R-HA(A/Anhui/1/2005), pCMV/R-HA(A/Indonesia/05/2005), and pCMV/R-HA(A/chicken/Nigeria/641/2006) in the dose response study at the indicated DNA vaccine doses were analyzed. Neutralization of A/Vietnam/1203/2004 or A/Indonesia/05/2005 HA was performed by LAI as described in the Materials and Methods. End point dilutions of the serum with IC_50_ activity are shown.
(0.11 MB DOC)

Figure S1 Characterization of needle-free (Agro-Jet®) DNA immunization in chickens. To evaluate the distribution of fluid into superficial or deep layers of subcutaneous tissues after delivery by AgroJet®, 4 or 7 week old chickens were injected with a solution containing India ink with this needle-free device at various pressures, ranging from 45 to 55 mm Hg. Three sites (thigh, wing and breast) were used, and biopsies were taken for routine hematoxylin and eosin staining. Representative sections of thigh injections are shown from 7 week old chickens and were similar at 4 weeks (data not shown). While the 48 mm Hg pressure deposited the injectate into the dermis/subcutaneous region (left), the higher pressure injections, 52 and 58 mm Hg, deposited the injectate into the subcutaneous and muscle layers (middle, right). 48 mm Hg consistently provided an optimal pressure to deposit the injectate into the dermis and subcutaneous tissue and was chosen for all AgroJet® immunizations.
(10.74 MB DOC)
