# Correction: Multivalent HA DNA Vaccination Protects against Highly Pathogenic H5N1 Avian Influenza Infection in Chickens and Mice

**DOI:** 10.1371/annotation/b8b66a84-4919-4a3e-ba3e-bb11f3853755

**Published:** 2008-09-18

**Authors:** Srinivas Rao, Wing-Pui Kong, Chih-Jen Wei, Zhi-Yong Yang, Martha Nason, Darrel Styles, Louis J. DeTolla, Aruna Panda, Erin M. Sorrell, Haichen Song, Hongquan Wan, Gloria C. Ramirez-Nieto, Daniel Perez, Gary J. Nabel

Dr. Aruna Panda was not included in the Author Byline. She should be listed as the eighth author. Please view the corrected author byline, with affiliations here:

Srinivas Rao^1^, Wing-Pui Kong^1^, Chih-Jen Wei^1^, Zhi-Yong Yang^1^, Martha Nason^1^, Darrel Styles^2^, Louis J. DeTolla^4^, Aruna Panda^4^, Erin M. Sorrell^3^, Haichen Song^3^, Hongquan Wan^3^, Gloria C. Ramirez-Nieto^3^, Daniel Perez^3^, Gary J. Nabel^1^

1 Vaccine Research Center, National Institute of Allergy and Infectious Diseases, National Institutes of Health, Bethesda, Maryland, United States of America, 2 United States Department of Agriculture, Animal and Plant Health Inspection Service, Riverdale, Maryland, United States of America, 3 College of Veterinary Medicine, University of Maryland, College Park, Maryland, United States of America, 4 Comparative Medicine, University of Maryland Baltimore, Baltimore, Maryland, United States of America

