# Correction: Multivalent HA DNA Vaccination Protects against Highly Pathogenic H5N1 Avian Influenza Infection in Chickens and Mice

**DOI:** 10.1371/annotation/5fbbf39a-fb47-4ce1-8069-acd830b3d41f

**Published:** 2008-09-18

**Authors:** Srinivas Rao, Wing-Pui Kong, Chih-Jen Wei, Zhi-Yong Yang, Martha Nason, Darrel Styles, Louis J. DeTolla, Aruna Panda, Erin M. Sorrell, Haichen Song, Hongquan Wan, Gloria C. Ramirez-Nieto, Daniel Perez, Gary J. Nabel

Because the author Aruna Panda was added to the author byline after publication, the Author Contributions section does not correctly reflect the new authorship. The new Author Contributions should read:

Conceived and designed the experiments: GN WK ZY DP SR CW DS. Performed the experiments: WK ZY DP LD SR CW ES HS HW GR AP. Analyzed the data: GN WK ZY DP MN SR CW DS. Wrote the paper: GN WK ZY DP SR CW DS AP.

