# Supplementary material for: Multivalent HA DNA Vaccination Protects against Highly Pathogenic H5N1 Avian Influenza Infection in Chickens and Mice
Source: PLoS One. 2008 Jun 18;3(6):e2432. doi: 10.1371/journal.pone.0002432 (PMC2657001; doi:10.1371/journal.pone.0002432)
Supplement: Table S1 — Hemagglutination inhibition (HI), microneutralization titer (NT), and LAI of sera from individual chickens immunized with different vaccines. Sera from immunized animals were obtained at week 5 or 7, a week before or after the final boost, and neutralization was assessed by HI, microneutralization (NT) and LAI (shown as IC50). Individual animal serum of each group is shown and was analyzed as described in the Materials and Methods section. (0.12 MB DOC) [file pone.0002432.s001.doc]

| Table S1 |  |  |  |  |  |  |  |
| --- | --- | --- | --- | --- | --- | --- | --- |
| **Immunogen** | **Animal ID** | **HI Titer** | | **NT Titer** | | **LAI Titer (VN1203)** | |
|  |  | **Week5** | **Week7** | **Week5** | **Week7** | **Week5** | **Week 7** |
| Agro-Jet x3 Control 500 µg | 202 | 40 | 40 | 10 | 10 | <100 | <100 |
|  | 203 | 40 | 40 | 10 | 10 | <100 | <100 |
|  | 204 | 20 | 20 | 10 | 10 | <100 | <100 |
|  | 205 | 40 | 20 | 10 | 10 | <100 | <100 |
|  | 206 | 40 | 20 | 10 | 10 | <100 | <100 |
|  | 207 | 20 | 20 | 10 | 10 | <100 | <100 |
|  | 208 | 40 | 40 | 10 | 10 | <100 | <100 |
|  | 209 | 40 | 20 | 10 | 10 | <100 | <100 |
| Agro-Jet x3 A/Indonesia/05/05  500 µg | 211 | 80 | 80 | 160 | 160 | 141 | 884 |
|  | 212 | 20 | 640 | 160 | 640 | 156 | >3200 |
|  | 213 | 40 | 80 | 40 | 160 | 452 | 806 |
|  | 214 | 20 | 160 | 20 | 160 | 396 | 670 |
|  | 215 | 80 | 40 | 40 | 320 | 696 | 1436 |
|  | 216 | 40 | 80 | 80 | 320 | 1284 | >3200 |
|  | 217 | 40 | 40 | 20 | 640 | 216 | >3200 |
|  | 218 | 20 | 40 | 40 | 160 | 159 | 1088 |
| Agro-Jet x3  A/Indonesia/05/05  A/Vietnam/1203/04  A/Anhui/01/05 500 µg | 233 | 40 | 640 | 40 | 640 | 381 | 1836 |
|  | 234 | 40 | 1280 | 40 | 1280 | 102 | 2911 |
|  | 235 | 20 | 1280 | 40 | 320 | <100 | >3200 |
|  | 236 | 40 | 1280 | 20 | 640 | 118 | 3013 |
|  | 237 | 20 | 1280 | 40 | 1280 | 237 | >3200 |
|  | 238 | 20 | 160 | 10 | 10 | <100 | <100 |
|  | 239 | 20 | 1280 | 20 | 640 | 145 | >3200 |
|  | 240 | 80 | 1280 | 40 | 640 | 127 | 2780 |
| Agro-Jet x2  A/Indoensia/05/05 500 µg | 242 | 40 | 40 | 40 | 10 | <100 | <100 |
|  | 243 | 40 | 80 | 40 | 10 | <100 | <100 |
|  | 244 | 40 | 80 | 40 | 10 | <100 | <100 |
|  | 245 | 40 | 1280 | 20 | 320 | 100 | 992 |
|  | 246 | 40 | 1280 | 40 | 320 | 190 | 2618 |
|  | 247 | 40 | 1280 | 40 | 1280 | <100 | 2679 |
|  | 248 | 40 | 10 | 40 | 20 | <100 | <100 |
|  | 249 | 80 | 640 | 80 | 640 | 183 | 2127 |
| sc N/S x3 A/Indonesia/05/05 500 µg | 251 | 80 | 20 | 20 | 40 | 180 | 161 |
|  | 252 | 80 | 80 | 320 | 320 | 531 | 764 |
|  | 253 | 80 | 80 | 40 | 160 | 213 | 857 |
|  | 254 | 40 | 160 | 1280 | 80 | 2233 | 985 |
|  | 255 | 320 | 320 | 1280 | 640 | 2564 | 2878 |
|  | 256 | 40 | 80 | 10 | 10 | 163 | 104 |
|  | 257 | 80 | 320 | 80 | 80 | 493 | 476 |
|  | 258 | 80 | 80 | 320 | 160 | 1269 | 650 |
| im N/S x3 A/Indonesia/05/05 500 µg | 259 | 40 | 80 | 1280 | 80 | 971 | 583 |
|  | 260 | 160 | 160 | 320 | 160 | 1521 | 864 |
|  | 261 | 160 | 160 | 640 | 80 | 1945 | 532 |
|  | 262 | 40 | 80 | 40 | 40 | 197 | 254 |
|  | 263 | 80 | 40 | 40 | 20 | 631 | 291 |
|  | 264 | 320 | 1280 | 640 | 160 | 2366 | 1688 |
|  | 265 | 80 | 1280 | 1280 | 320 | 2132 | 1539 |
|  | 266 | 40 | 80 | 80 | 80 | 416 | 394 |
