# Supplementary material for: Multivalent HA DNA Vaccination Protects against Highly Pathogenic H5N1 Avian Influenza Infection in Chickens and Mice
Source: PLoS One. 2008 Jun 18;3(6):e2432. doi: 10.1371/journal.pone.0002432 (PMC2657001; doi:10.1371/journal.pone.0002432)
Supplement: Table S2 — Neutralizing antibody responses after two vaccinations at different doses by LAI. Sera obtained at week 5, one week after the final boost, from individual animals immunized with trivalent DNA HA encoding vaccine: pCMV/R-HA(A/Anhui/1/2005), pCMV/R-HA(A/Indonesia/05/2005), and pCMV/R-HA(A/chicken/Nigeria/641/2006) in the dose response study at the indicated DNA vaccine doses were analyzed. Neutralization of A/Vietnam/1203/2004 or A/Indonesia/05/2005 HA was performed by LAI as described in the Materials and Methods. End point dilutions of the serum with IC50 activity are shown. (0.11 MB DOC) [file pone.0002432.s002.doc]

Table S2

| **Immunogen** | **LAI Titer (Week5)** | | | | | |
| --- | --- | --- | --- | --- | --- | --- |
|  | **Agro-Jet®** | | | **Needle & Syringe** | | |
|  | **Animal ID** | **VN/1203** | **IN/05/05** | **Animal ID** | **VN/1203** | **IN/05/05** |
| 500µg Control | 46 | <100 | <100 | 27 | 102 | 109 |
|  | 47 | <100 | <100 | 28 | <100 | <100 |
|  | 48 | <100 | <100 | 29 | <100 | <100 |
|  | 49 | <100 | <100 | 268 | <100 | <100 |
|  | 289 | <100 | <100 | 269 | 150 | 134 |
|  | 290 | <100 | <100 | 270 | <100 | <100 |
|  | 291 | <100 | <100 | 271 | <100 | <100 |
|  | 292 | <100 | <100 |  |  |  |
| 500µg A/Indonesia/05/05 A/Anhui/01/05 A/c/Nigeria/641/05 | 52 | <100 | 163 | 30 | 125 | 167 |
|  | 53 | 258 | 600 | 31 | <100 | 115 |
|  | 54 | 209 | 510 | 32 | <100 | 142 |
|  | 55 | 434 | 1360 | 33 | <100 | <100 |
|  | 293 | <100 | <100 | 272 | 169 | 181 |
|  | 294 | <100 | 341 | 273 | <100 | 142 |
|  | 295 | <100 | 122 | 274 | <100 | <100 |
|  | 296 | 114 | 378 | 275 | 194 | 614 |
| 50µg A/Indonesia /05/05 A/Anhui/01/05 A/c/Nigeria/641/05 | 56 | 258 | 547 | 34 | <100 | 131 |
|  | 57 | <100 | 225 | 35 | <100 | 145 |
|  | 58 | 504 | 2285 | 36 | <100 | <100 |
|  | 59 | 119 | 405 | 37 | <100 | <100 |
|  | 297 | 139 | 1019 | 277 | <100 | <100 |
|  | 298 | 297 | 696 | 278 | <100 | 140 |
|  | 299 | 337 | 2060 | 279 | <100 | <100 |
|  | 300 | <100 | 122 | 280 | <100 | <100 |
| 5µg A/Indonesia /05/05 A/Anhui/01/05 A/c/Nigeria/641/05 | L47 | <100 | 152 | 38 | 125 | 149 |
|  | L67 | <100 | <100 | 39 | <100 | <100 |
|  | L69 | 322 | 1526 | 40 | <100 | <100 |
|  | L71 | 271 | 1284 | 41 | <100 | <100 |
|  | 78 | <100 | 819 | 281 | 144 | 111 |
|  | 79 | <100 | <100 | 282 | <100 | <100 |
|  | 80 | <100 | 819 | 283 | <100 | <100 |
|  | 81 | <100 | <100 | 284 | <100 | <100 |
| 0.5µg A/Indonesia /05/05 A/Anhui/01/05 A/c/Nigeria/641/05 | L72 | <100 | <100 | 43 | <100 | <100 |
|  | L73 | <100 | 130 | 44 | <100 | <100 |
|  | L74 | <100 | <100 | 50 | <100 | <100 |
|  | L75 | <100 | <100 | 285 | <100 | <100 |
|  | L99 | 157 | 908 | 286 | <100 | <100 |
|  | 26 | <100 | <100 | 287 | <100 | <100 |
|  | 82 | <100 | <100 | 288 | <100 | <100 |
|  | 96 | <100 | <100 |  |  |  |
