# Supplementary figures and images for: Multivalent HA DNA Vaccination Protects against Highly Pathogenic H5N1 Avian Influenza Infection in Chickens and Mice
Source: PLoS One. 2008 Jun 18;3(6):e2432. doi: 10.1371/journal.pone.0002432 (PMC2657001; doi:10.1371/journal.pone.0002432)

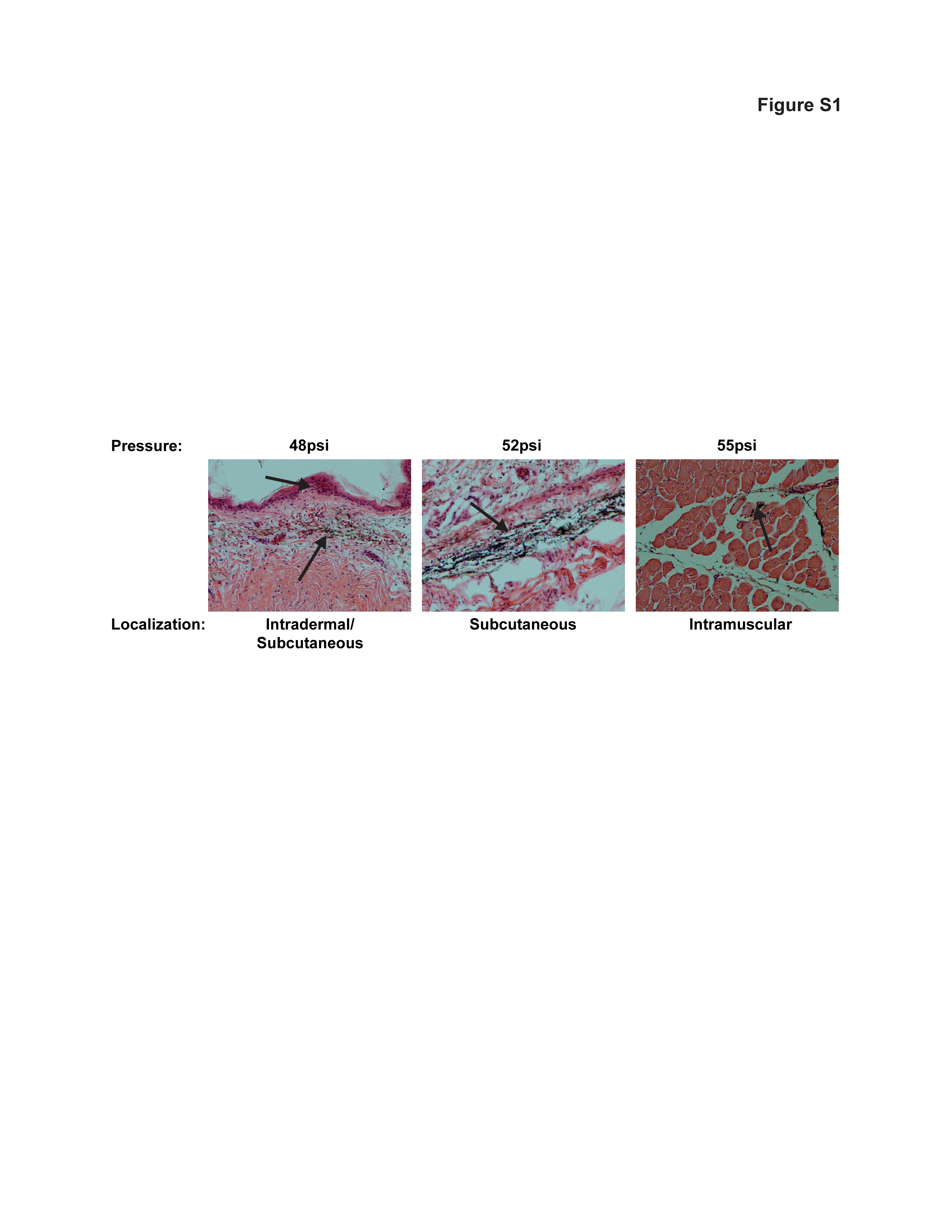

Supplement: Figure S1 — Characterization of needle-free (Agro-Jet®) DNA immunization in chickens. To evaluate the distribution of fluid into superficial or deep layers of subcutaneous tissues after delivery by AgroJet®, 4 or 7 week old chickens were injected with a solution containing India ink with this needle-free device at various pressures, ranging from 45 to 55 mm Hg. Three sites (thigh, wing and breast) were used, and biopsies were taken for routine hematoxylin and eosin staining. Representative sections of thigh injections are shown from 7 week old chickens and were similar at 4 weeks (data not shown). While the 48 mm Hg pressure deposited the injectate into the dermis/subcutaneous region (left), the higher pressure injections, 52 and 58 mm Hg, deposited the injectate into the subcutaneous and muscle layers (middle, right). 48 mm Hg consistently provided an optimal pressure to deposit the injectate into the dermis and subcutaneous tissue and was chosen for all AgroJet® immunizations. (10.74 MB DOC) [file pone.0002432.s003.tif]
